# Pulsed Electromagnetic Fields Improve Bone Microstructure and Strength in Ovariectomized Rats through a Wnt/Lrp5/β-Catenin Signaling-Associated Mechanism

**DOI:** 10.1371/journal.pone.0079377

**Published:** 2013-11-14

**Authors:** Da Jing, Feijiang Li, Maogang Jiang, Jing Cai, Yan Wu, Kangning Xie, Xiaoming Wu, Chi Tang, Juan Liu, Wei Guo, Guanghao Shen, Erping Luo

**Affiliations:** 1 Department of Biomedical Engineering, Fourth Military Medical University, Xi’an, China; 2 Department of Endocrinology, Xijing hospital, Fourth Military Medical University, Xi’an, China; 3 Institute of Orthopaedics, Xijing hospital, Fourth Military Medical University, Xi’an, China; National Research Council, Italy

## Abstract

Growing evidence has demonstrated that pulsed electromagnetic field (PEMF), as an alternative noninvasive method, could promote remarkable *in vivo* and *in vitro* osteogenesis. However, the exact mechanism of PEMF on osteopenia/osteoporosis is still poorly understood, which further limits the extensive clinical application of PEMF. In the present study, the efficiency of PEMF on osteoporotic bone microarchitecture and bone quality together with its associated signaling pathway mechanisms was systematically investigated in ovariectomized (OVX) rats. Thirty rats were equally assigned to the Control, OVX and OVX+PEMF groups. The OVX+PEMF group was subjected to daily 8-hour PEMF exposure with 15 Hz, 2.4 mT (peak value). After 10 weeks, the OVX+PEMF group exhibited significantly improved bone mass and bone architecture, evidenced by increased BMD, Tb.N, Tb.Th and BV/TV, and suppressed Tb.Sp and SMI levels in the MicroCT analysis. Three-point bending test suggests that PEMF attenuated the biomechanical strength deterioration of the OVX rat femora, evidenced by increased maximum load and elastic modulus. RT-PCR analysis demonstrated that PEMF exposure significantly promoted the overall gene expressions of Wnt1, LRP5 and β-catenin in the canonical Wnt signaling, but did not exhibit obvious impact on either RANKL or RANK gene expressions. Together, our present findings highlight that PEMF attenuated OVX-induced deterioration of bone microarchitecture and strength in rats by promoting the activation of Wnt/LRP5/β-catenin signaling rather than by inhibiting RANKL-RANK signaling. This study enriches our basic knowledge to the osteogenetic activity of PEMF, and may lead to more efficient and scientific clinical application of PEMF in inhibiting osteopenia/osteoporosis.

## Introduction

Osteoporosis is one of the most common diseases in clinics, characterized by significant bone mass loss and bone microarchitecture deterioration, resulting in bone fragility and an increased risk of fractures. Osteoporosis has become a leading cause of mortality and morbidity and exerts tremendous economic and social burdens to both developed countries and developing countries [1]. Traditional pharmacological agents either promoting bone formation (e.g., parathyroid hormone, insulin-like growth factor and growth hormone) or inhibiting bone resorption (e.g., calcitonin, estrogen and bisphosphonate) may help partially prevent and reverse osteoporosis, but the high cost and potential side effects might become a non-negligible limitation [2], [3], [4]. Therefore, it is essential to develop more economic and safe non-pharmaceutical approaches for the prevention and treatment of osteoporosis, which can benefit most populations in developing countries.

Since the first application of pulsed electromagnetic fields (PEMF) by Basset et al. in accelerating clinical bone fracture healing in 1974 [5], [6], the biological effects of PEMF have gained extensive attention. In the past four decades, substantial and growing evidence has accumulated to show that PEMF therapy as an alternative noninvasive method is capable of producing satisfying therapeutic effects on a wide range of bone diseases, such as fresh and nonunion fractures [7], [8] and osteoarthritis [9], [10], *etc*. Several experimental studies have demonstrated that PEMF stimulation could promote potently osteogenesis and enhance bone mineralization both *in vivo* and *in vitro* [11], [12], [13], [14], [15], [16], [17]. Clinical investigations further revealed that PEMF could help enhance bone mineral density (BMD) and reduce the risk of fractures in osteoporotic patients [18], [19], [20]. Despite of these positive findings, the exact signaling pathways and their associated regulatory mechanisms of PEMF therapy on osteopenia/osteoporosis are still unclear, which might further limit the extensive clinical application of PEMF.

Numerous studies have proved that the canonical Wnt signaling (i.e., Wnt/LRP5/β-catenin) and RANKL-RANK signaling are two of the most essential pathways mediating bone metabolism and bone quality [21], [22]. First, Wnts, as a family of secreted proteins existing extensively within the skeleton, are able to regulate bone formation via multiple routes, including promoting the differentiation of mesenchymal stem cells into mature osteoblasts, enhancing the proliferation and mineralization of osteoblasts, and preventing the apoptosis of osteoblasts and osteocytes [23]. Second, the receptor activator of NF-κB (RANK) and its ligand RANKL have been proved to function to be a critical and central mediator for osteoclast growth, maturation and activation [24]. RANKL acts as a negative regulator of bone mass and bone quality, and overexpression of RANKL in bone can result in significant bone loss [25], [26]. Therefore, understanding the roles of canonical Wnt signaling and RANKL-RANK signaling in preventing osteopenia/osteoporosis by PEMF carries great significance for deciphering the mechanisms by which bone processes the PEMF stimulus. To date, however, this issue is still poorly understood.

In the present study, the inhibitive effects of PEMF stimulation on bone microstructure deterioration and bone mechanical strength decrease were systematically evaluated in ovariectomy (OVX)-induced osteoporotic rats via MicroCT analysis and biomechanical testing. Moreover, the relevant signaling pathway mechanisms of PEMF exposure, including Wnt/LRP5/β-catenin signaling and RANKL-RANK signaling pathways, were also systematically investigated.

## Materials and Methods

### Animals

Thirty 3-month-old female Sprague-Dawley rats (270±15.0 g) obtained from the Animal Center of the Fourth Military Medical University were used in this study. Rats were housed under controlled standard temperature (23±1°C), relative humidity (50% ∼ 60%) and 12∶12 h light-dark cycle (light on from 7 a.m. to 7 p.m.). All animals were allowed ad libitum access to clean tap water and standard rodent pelleted chow (Animal Center of the Fourth Military Medical University, Xi’an, China) throughout the experimental period. All procedures in the experiment were in strict accordance with the guiding principles of Institutional Animal Ethical Committee (IAEC), Committee for the Purpose of Control and Supervision of Experiments on Animals (CPCSEA), and the Guide for the Care and Use of Laboratory Animals published by the National Institutes of Health [NIH Publication.85-23]. The animal protocol was approved by the Institutional Animal Care and Use Committee of the Fourth Military Medical University (Permit Number 11427). All efforts were made to minimize the number of animals used and their suffering. All surgery was performed under diethyl ether anesthesia.

### Experimental Design

All animals were divided into three equal groups (*n* = 10) and randomly assigned to the sham-operated control group (Control), ovariectomy group (OVX), and OVX with PEMF exposure group (OVX+PEMF). After ether anesthetization, rats were subjected to dorsal bilateral OVX or sham operation. A single longitudinal incision was created on the midline of the dorsal skin at the level of both kidneys. For the rats in OVX and OVX+PEMF groups, both ovaries were ligated and removed using surgical suture. Rats in the Control group were subjected to sham surgery and both ovaries were exposed but maintained intact to eliminate the effects of the surgery. Rats received daily intramuscular injection with penicillin to avoid the infection of pathogens. Each rat was housed separately in one single cage within 3 days post surgery to avoid any potential cross infection. Rats were housed in six custom-designed plastic cages (400 mm×240 mm×240 mm) with 5 rats in each cage on the 4^th^ post-operative day. Rats in the OVX+PEMF group were subjected to whole-body PEMF exposure with 8 hours per day (PEMF on from 8 a.m. to 4 p.m.) for 10 weeks. The cages of Control and OVX groups were also placed in the PEMF coil assembly, but the coils were inactivated to provide sham PEMF stimulation. Animals were inspected daily for signs of any disease and weighed weekly throughout the experimental period. At the end of 10-week PEMF exposure, rats were euthanatized with an overdose of diethyl ether. The left and right femora were harvested, wrapped in saline-soaked gauze and stored at −20°C, which were used for mechanical testing and MicroCT analysis, respectively. Fresh bilateral tibiae were harvested for RT-PCR analysis.

### PEMF Treatment

**Figure 1:**
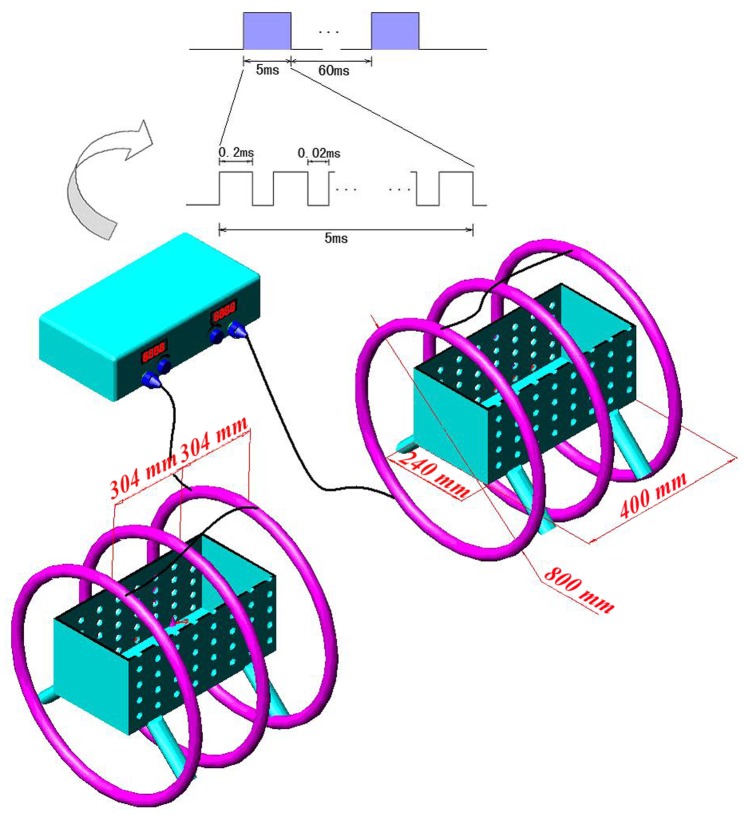
Schematic representation of the PEMF generator together with a Helmholz coil assembly with three-coil array[14]. The PEMF output waveform consisted of a pulsed burst (burst width, 5 ms; pulse width, 0.2 ms; pulse wait, 0.02 ms; burst wait, 60 ms; pulse rise, 0.3 µs; pulse fall, 2.0 µs) repeated at 15 Hz. The peak magnetic field intensity within the Helmholtz coils was calculated to be approximately 2.4 mT.

As shown in **Fig. 1** [14], the PEMF exposure device (GHY-III, FMMU, Xi’an, China; China Patent no.ZL02224739.4) was composed of a pulsed signal generator and a Helmholz coil assembly with three-coil array. The coils were made up of enameled coated copper wire with 0.8 mm diameter and placed coaxially with a distance of 304 mm apart from each other. The magnetic field value along the O-X direction (axial direction of the coil) is expressed by:
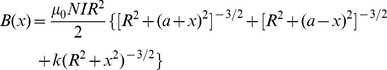
where *µ_0_* is the permeability of vacuum, *I* is the current through the coils, *R* is the radius of the coils, *a* is the distance between the central coil and the outside coil, *x* is the abscissa relative to origin (center of the middle coil), *N* is the number of turns of the outside coil, and *K·N* is the number of turns of the middle coil. By setting the parameters 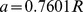 and 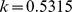, the second and fourth derivative of *B(x)* will become zero at the position of origin and then the maximum uniformity of the magnetic field extent will be obtained [27]. Therefore, we obtain the number of turns of the central coil, 266 turns, and the number of turns of the outside coils, 500 turns, and the distance between the central coil and the outside coil, approximately 304 mm. The relative deviation of magnetic field intensity between at any point on the abscissa axis and the origin is: 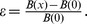 The numerical calculation by Wang et al. demonstrated that this improved HelmHolz coil with three-coil array displayed significantly upgraded axial magnetic field uniformity (for 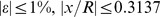 in HelmHolz coil with two-coil array and 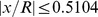 in improved HelmHolz coil), as well as decreased deviation of the magnetic field between the origin and any other off-axial point within the coils [27].

The cage was held up by a plastic support with four legs of which height could be freely adjusted. The bottom of the cage was aligned with the center of the coils to ensure that the rats were confined in the center of the electromagnetic fields where the flux density was considered to be uniform. The PEMF waveform consisted of a pulsed burst (burst width, 5 ms; burst wait, 60 ms; burst duty circle, 7.7%; pulse width, 0.2 ms; pulse wait, 0.02 ms; pulse rise, 0.3 µs; pulse fall, 2.0 µs; pulse duty circle, 90.0%) repeated at 15 Hz. A small resistor of 2 Ω was placed in series with the coils and the voltage drop across the resistor was observed with an Agilent 6000 Series oscilloscope (Agilent Technologies, Santa Clara, CA, USA). The peak voltage drop was observed to calculate the peak current in the coils. The peak magnetic field of the coils in the OVX+PEMF group was determined as approximately 2.4 mT. The accuracy for the peak magnetic field measurement was further confirmed by using a Gaussmeter (Model 455 DMP Gaussmeter, Lake Shore Cryotronics, USA) and the root mean square (RMS) value of the magnetic field was determined to be approximately 0.5 mT. This waveform has been shown to generate positive osteogenetic effects by our research group for a long period of experiment [13], [14]. The measured background electromagnetic field was 0.5±0.02 Gs.

### Biomechanical Examination

The frozen left femora in saline soaked gauze were thawed in physiological saline solution at room temperature for 1 h before three-point bending test. The biomechanical properties of the femora were evaluated using a commercial mechanical testing device (AGS*-*10kNG, Shimadzu, Kyoto, Japan). The sample was submerged in physiological saline solution until undergoing mechanical testing. The femur with its physiological curvature facing up was stabilized on a sample supporter with two fixed loading points with 20-mm interval distance. The upper loading plate was oriented perpendicularly to the long axis of the femur, and the upper loading point was located at the midpoint between the two lower loading points. A preload with 2 N was applied to immobilize the sample prior to the mechanical testing. Then, load was applied at a constant displacement rate of 2 mm/min by controlling the motion of the upper loading plate until bone fracture occurred. The load-displacement curve was simultaneously plotted and data were automatically recorded into a computer which was interfaced to the material testing machine. After mechanical testing, the internal and external major axis and minor axis lengths of the femur at the fracture point were immediately measured using a vernier caliper. The following parameters could be directly obtained from the load-displacement curve, including maximum load (maximum force that the femur can sustain before failure), stiffness (slope of the linear part of the load-displacement curve representing elastic deformation), and energy absorption (area under the load-deformation curve until failure). Elastic modulus was calculated according to the formula [28]: 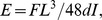 where *F* is the maximum load, *L* is the distance between the two lower supporting points, *d* is the displacement, *I* is the moment of inertia of the cross-section in relation to the horizontal axis of the femur.

### MicroCT Analysis

The right femora were thawed at room temperature for 1 h and bone microarchitecture was evaluated using a high-resolution MicroCT (GE healthcare, Madison, WI, USA). The basic scanning parameters were set as the following: voltage 80 kV, current 80 µA, exposure time 2.96 s, total rotation angle 210^o^, and rotation angle of increment 0.4^o^. The scanning resolution was 16 µm/slice. The distal femur was placed in a 20-mm-diameter sample tube perpendicularly to the scanning axis with a total 12-mm reconstruction height. It took approximately 30 min for the scanning. The image sequences were transferred to a workstation after scanning, and 3-D reconstruction was performed for image visualization and data analysis. A volume of interest (VOI) with 1.6-mm height was selected for the analysis of trabecular bone microarchitecture. The VOI started at a distance of 0.4 mm (25 slices) from the lowest end of the growth plate and extended to the proximal end of the femur with a distance of 1.6 mm (100 slices). This VOI selection could help avoid measuring the primary spongiosa and only contain the secondary spongiosa. The trabecular bone microarchitecture could be visualized and quantitatively analyzed with the MicroView program (GE healthcare, Madison, WI, USA). The quantitative analysis of trabecular bone microarchitecture included the following indices: trabecular BMD, trabecular number (Tb.N), trabecular thickness (Tb.Th), trabecular separation (Tb.Sp), bone volume per tissue volume (BV/TV), and structure model index (SMI).

### RNA Extraction and RT-PCR

Fresh bilateral tibiae were harvested and cleaned with cold PBS. After removal of bone marrow, bone samples were immediately crushed into powder using the pestle and then mixed with the monophasic solution of phenol and guanidine thiocyanate. Total RNA was extracted using the guanidinium isothiocyanate-alcohol phenyl-chloroform method according to the instruction of the manufacturer. Then, SuperScript III reverse transcriptase was used to synthesize cDNA from RNA. Polymerase chain reaction (PCR) was performed according to 2× Taq PCR MasterMix reaction system. The primers were synthesized by Nanjing Genscript Biological Engineering Technology & Service Co., Ltd., China. The primers used in this study was specified as following: GGGGAGCAACCAAAGTCG (forward) and TGGAGGAGGCTATGTTCACG (reverse) for Wnt1, GACATTTACTGGCCCAATGG (forward) and CTGCCCTCCACCACCTTCT (reverse) for LRP5, GGAAAGCAAGCTCATCATTCT (forward) and AGTGCCTGCATCCCACCA (reverse) for β-catenin, ACGCAGATTTGCAGGACTCGAC (forward) and TTCGTGCTCCCTCCTTTCATC (reverse) for RANKL, TTAAGCCAGTGCTTCACGGG (forward) and ACGTAGACCACGATGATGTCGC (reverse) for RANK, and GCCAACACAGTGCTGTCT (forward) and AGGAGCAATGATCTTGATCTT (reverse) for β-actin. Wnt1, LRP5, β-catenin, RANKL and RANK mRNA expressions were normalized by the housekeeping gene β-actin.

### Statistical Analysis

All data presented in this study were expressed as the mean ± standard deviation (S.D.). Statistical analyses were performed using Microsoft SPSS version 13.0 for Windows software (SPSS, Chicago, IL, USA). One-way analysis of variance (ANOVA) was employed for evaluating the existence of differences among the three groups and once a significant difference was detected, Fisher’s least significant difference (LSD) t-test was used to determine the significance between every two groups. *P*<0.05 was considered statistically significant.

## Results

### Body Weight Measurement

**Figure 2:**
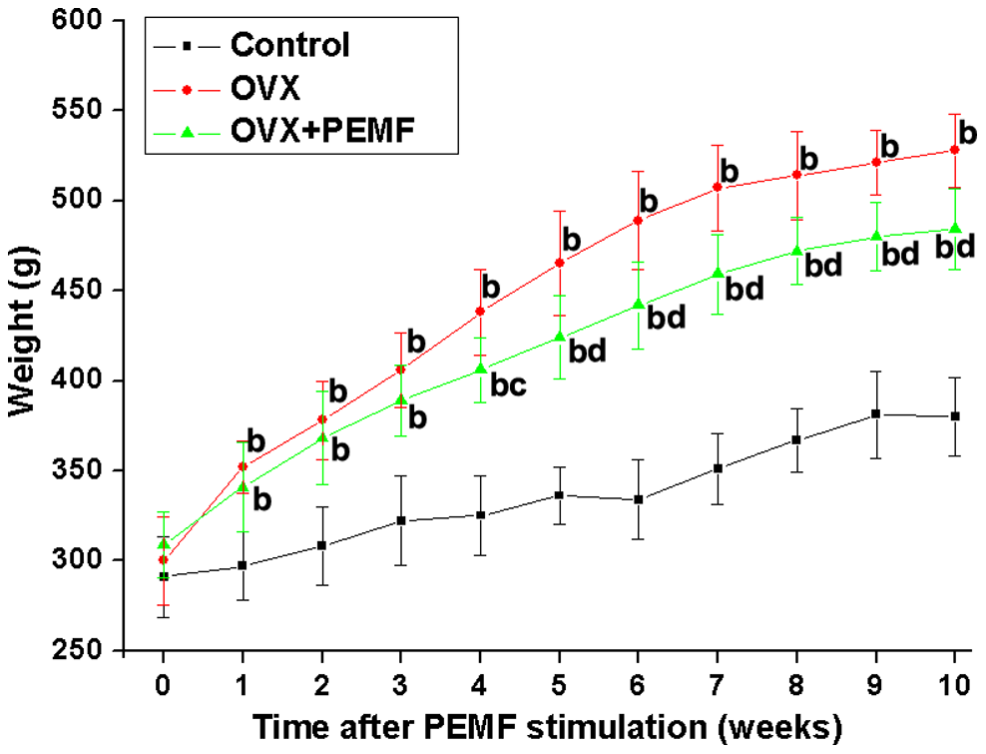
Effects of PEMF exposure on the weekly average body weights of OVX rats throughout the 10-week experimental period. Control, sham-operated control group; OVX, ovariectomy group; OVX+PEMF, ovariectomy with PEMF exposure group. Values are all expressed as mean ± S.D. (*n* = 10). ^b^Significant difference from the Control group with *P*<0.01; ^bc^Significant difference from the Control group with *P*<0.01 and OVX group with *P*<0.05; ^bd^Significant difference from the Control group and OVX group with *P*<0.01.

The weekly average body weights of rats in each group throughout the experimental period are shown in **Fig. 2**. Before PEMF stimulation, the body weights of rats in Control, OVX and OVX+PEMF groups were 289.6±24.7 g, 300.8±26.1 g and 309.2±21.4 g, respectively. During the 10-week experimental period, the body weights of rats in OVX and OVX+PEMF groups were significantly higher than those in the Control group (*P*<0.01). Furthermore, PEMF stimulation partially inhibited OVX-induced weight gain from the 4^th^ week (*P*<0.05).

### Biomechanical Examination

**Figure 3:**
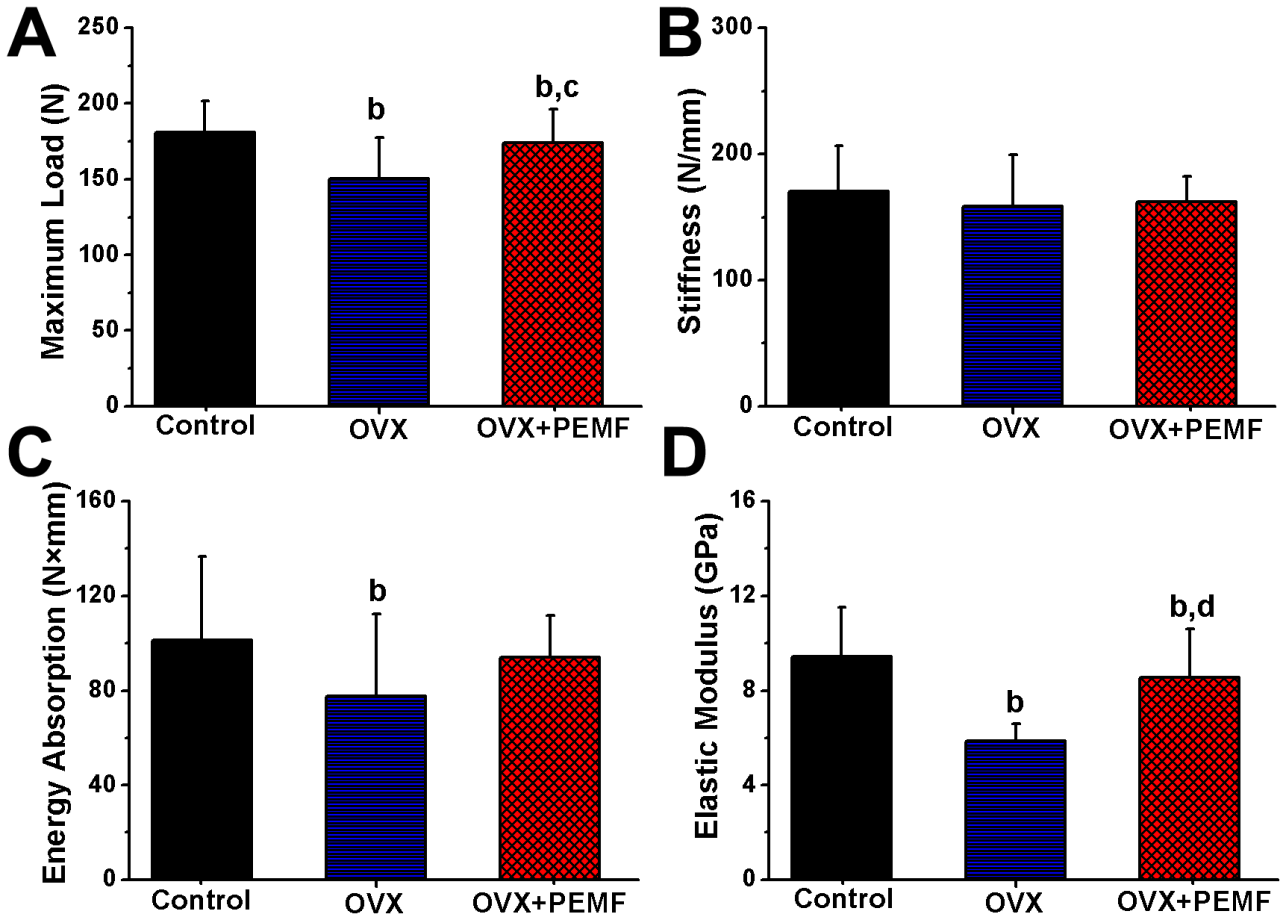
Effects of 10-week PEMF exposure on femoral biomechanical parameters (A) maximum load (B) stiffness (C) energy absorption (D) elastic modulus in OVX rats. Control, sham-operated control group; OVX, ovariectomy group; OVX+PEMF, ovariectomy with PEMF exposure group. Values are all expressed as mean ± S.D. (*n* = 10). ^b^Significant difference from the Control group with *P*<0.01; ^bc^Significant difference from the Control group with *P*<0.01 and OVX group with *P*<0.05; ^bd^Significant difference from the Control group and OVX group with *P*<0.01.

The examination results of biomechanical three-point bending experiments are shown in **Fig. 3**. OVX led to significant decreases in the maximum load and energy absorption as compared with the Control group (*P*<0.01). PEMF stimulation significantly increased the maximum load (*P*<0.05) of the femora in OVX rats. However, no significant difference in the stiffness of the femora was observed among the three groups. The femoral cross-sectional moment of inertia in the OVX group (4.64±0.61 mm^4^) was significantly higher than that in the Control group (3.07±0.57 mm^4^) and OVX+PEMF group (3.11±0.42 mm^4^) (*P*<0.01). Furthermore, OVX caused dramatic decrease of elastic modulus (*P*<0.01), which was significantly reversed after PEMF stimulation (*P*<0.01).

### MicroCT Analysis

**Figure 4:**
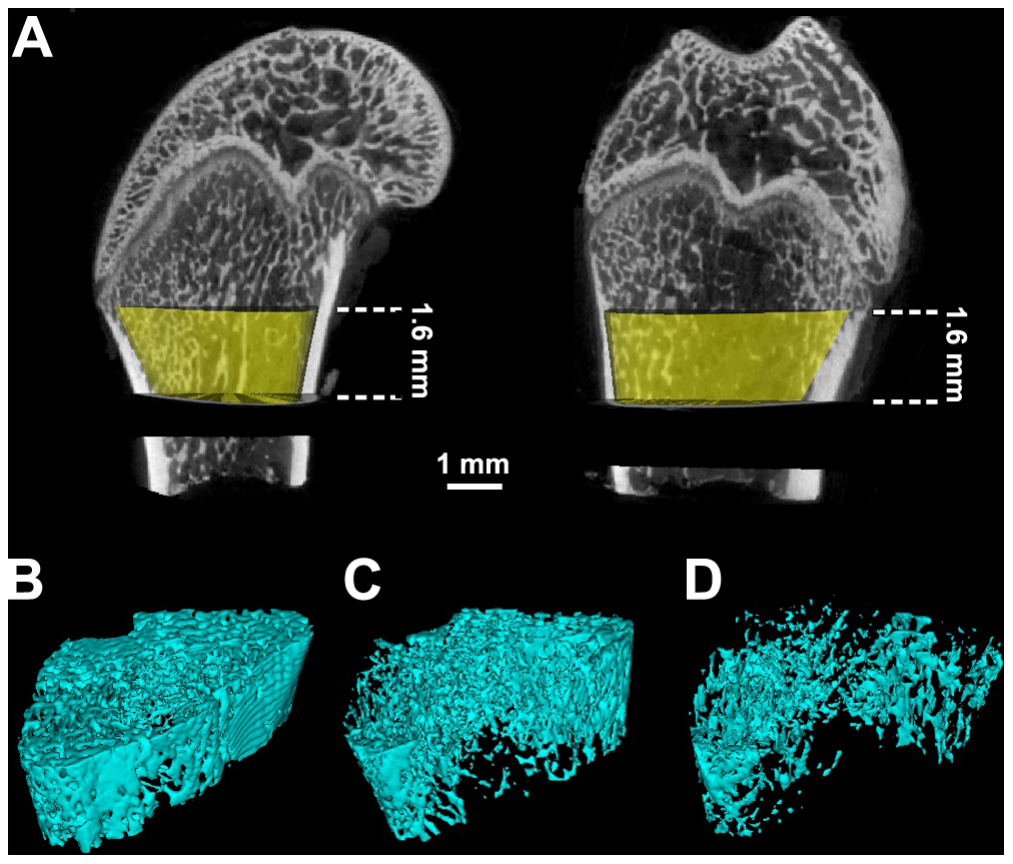
3-D MicroCT images of trabecular bone microarchitecture in the distal femora in Control, OVX and OVX+PEMF rats. A volume of interest (VOI) with 1.6 mm height was selected for the analysis of trabecular bone microarchitecture, which is represented with yellow color in **Fig. 4A**. The VOI started at a distance of 0.4 mm (25 slices) from the lowest end of the growth plate and extended to the proximal end of the femur with a distance of 1.6 mm (100 slices). Representative 3-D MicroCT images of femoral trabecular bone microarchitecture determined by the VOI were shown in **Fig. 4B∼D**. (**Fig. 4B**): Control group; (**Fig. 4C**): OVX+PEMF group; (**Fig. 4D**): OVX group. The femur in the OVX group exhibited significant decrease in the trabecular number, trabecular connectivity and trabecular area as compared with that in the Control group. PEMF exposure partially inhibited OVX-induced trabecular bone loss and significantly improved trabecular bone mass and bone microarchitecture.

**Figure 5:**
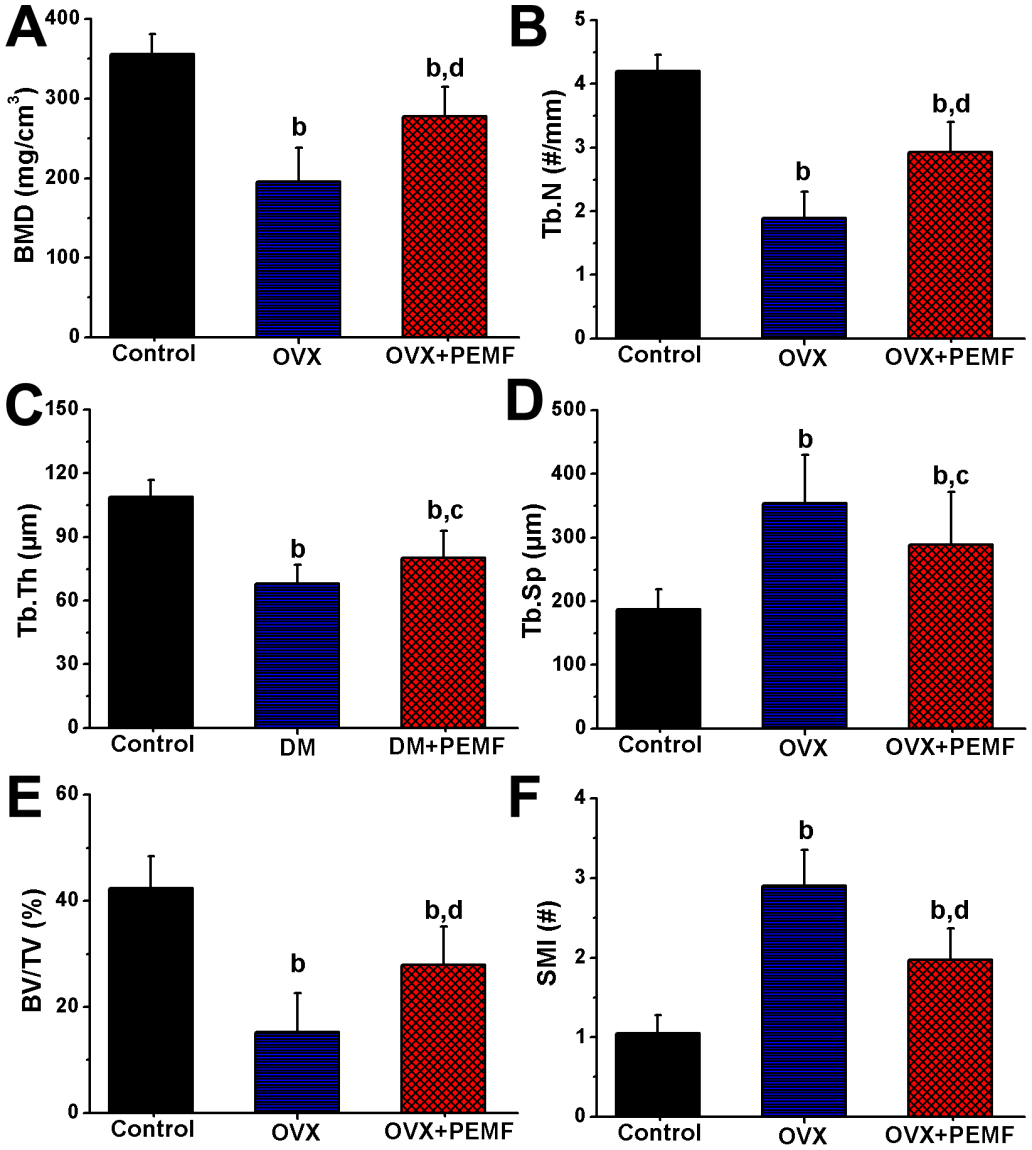
Effects of 10-week PEMF exposure on femoral trabecular MicroCT indices in OVX rats, including (A) bone mineral density (BMD), (B) trabecular number (Tb.N), (C) trabecular thickness (Tb.Th), (D) trabecular separation (Tb.Sp), (E) bone volume per tissue volume (BV/TV) and (F) structure model index (SMI). Control, sham-operated control group; OVX, ovariectomy group; OVX+PEMF, ovariectomy with PEMF exposure group; Values are all expressed as mean ± S.D. (*n* = 10). ^b^Significant difference from the Control group with *P*<0.01; ^ad^Significant difference from the Control group with *P*<0.05 and OVX group with *P*<0.01.

Representative 3-D MicroCT images demonstrating the inhibitive effects of PEMF on OVX-induced trabecular bone mass loss and bone microarchitecture deterioration are shown in **Fig. 4**. The femora from the OVX group displayed notable reduction in the trabecular number, trabecular connectivity and trabecular area as compared with those from the Control group. However, PEMF partially prevented OVX-induced bone loss and significantly improved the trabecular bone mass and bone microarchitecture. The statistical results for the femoral trabecular MicroCT analysis are shown in **Fig. 5**. OVX resulted in significant decrease in BMD, Tb.N, Tb.Th and BV/TV (*P*<0.01), and increase in Tb.Sp and SMI (*P*<0.01). Moreover, PEMF exposure dramatically reversed OVX-induced reduction in BMD (*P*<0.01), Tb.N (*P*<0.01), Tb.Th (*P*<0.05) and BV/TV (*P*<0.01), and also prevented OVX-induced increase in Tb.Sp (*P*<0.05) and SMI (*P*<0.01).

### RT-PCR Analysis

**Figure 6:**
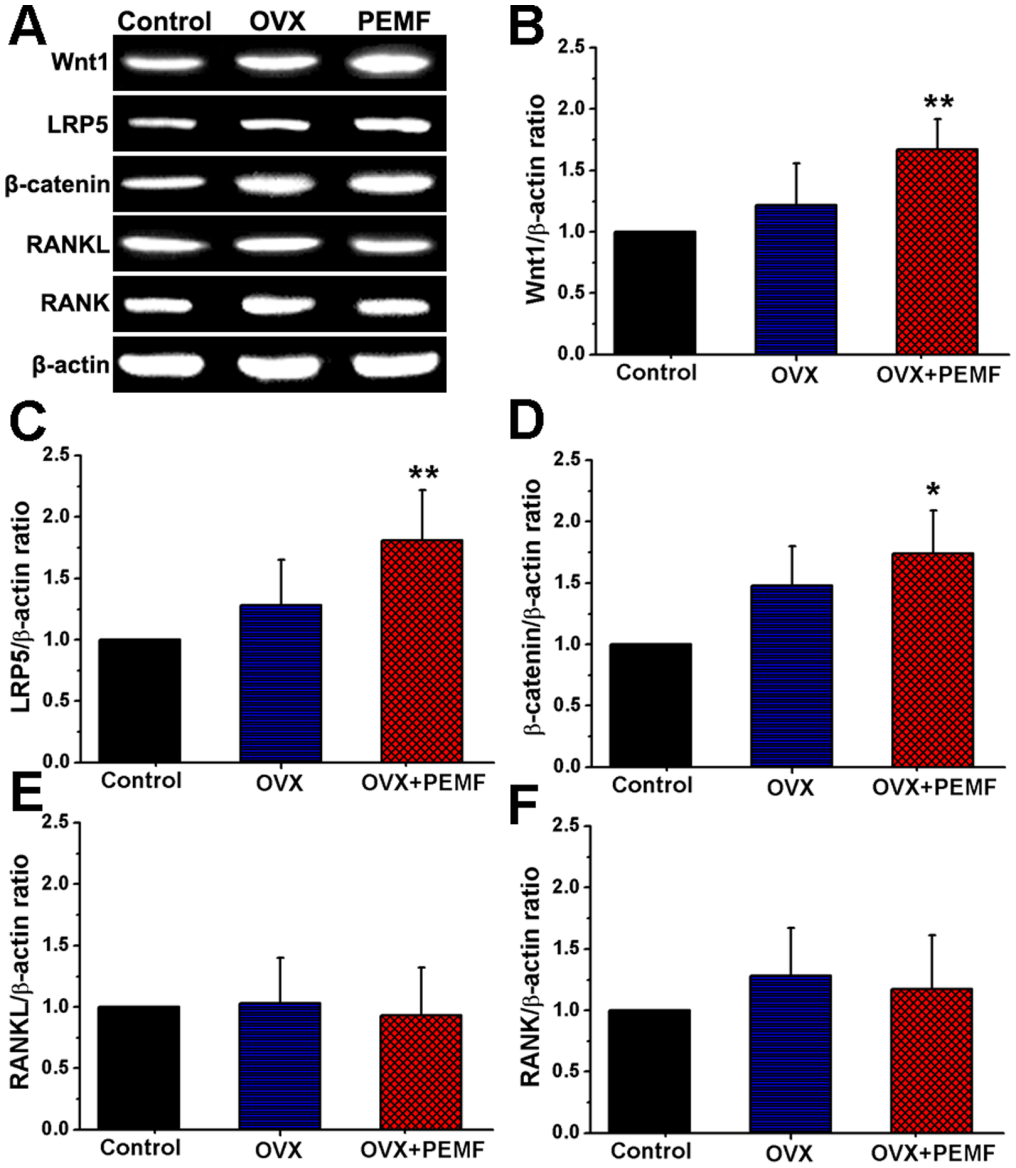
Effects of 10-week PEMF exposure on tibial Wnt1, LRP5, β-catenin, RANKL, RANK total mRNA expressions in OVX rats by RT-PCR analysis. (**A**) representative RT-PCR images for Wnt1, LRP5, β-catenin, RANKL, RANK and β-actin expressions, (**B**) Wnt1/β-actin ratio (*n* = 10), (**C**) LRP5/β-actin ratio (*n* = 10), (**D**) β-catenin/β-actin ratio (*n* = 10), (**E**) RANKL/β-actin ratio (*n* = 10) and (**F**) RANKL/β-actin ratio (*n* = 10) in rat tibiae of Control, OVX and OVX+PEMF groups. Control, sham-operated control group; OVX, ovariectomy group; OVX+PEMF, ovariectomy with PEMF exposure group; Values are all expressed as mean ± S.D. ^**^Significant difference from the OVX group with *P*<0.01.

The RT-PCR examination results for Wnt1, LRP5, β-catenin, RANKL, RANK and β-actin total mRNA expressions in Control, OVX and OVX+PEMF groups are depicted in **Fig. 6**. As shown in **Fig. 6A**, the mRNA gene expressions of Wnt1, LRP5, β-catenin and RANK in the OVX group were higher than those in the Control group, but RANKL gene expression did not show obvious difference between Control and OVX groups. PEMF stimulation caused remarkable increase of Wnt1, LRP5 and β-catenin gene expressions, but did not show obvious effects on RANKL and RANK mRNA expressions. The statistical results for Wnt1, LRP5, β-catenin, RANKL and RANK gene expressions (**Fig. 6B** **∼F**) further demonstrate that PEMF stimulation significantly increased the Wnt1/β-actin ratio, LRP5/β-actin ratio and β-catenin/β-actin ratio (*P*<0.01). However, PEMF exposure did not display significant effects on the levels of RANKL/β-actin ratio and RANK/β-actin ratio.

## Discussion

PEMF have proven to be an effective noninvasive method in the treatment of a wide range of bone diseases in clinics for several decades (e.g., nonunion fractures, osteoarthritis and osteoporosis). However, few experimental data to date have reported the effects of PEMF on osteoporotic bone microarchitecture and bone quality together with their associated signaling pathway mechanisms. Our findings in the present study clearly demonstrate that 10-week PEMF exposure remarkably attenuated OVX-induced bone mass loss and deterioration of bone microarchitecture and mechanical properties in rats by promoting overall gene expressions of Wnt/LRP5/β-catenin signaling rather than by inhibiting RANKL-RANK signaling.

According to our observations, OVX led to significant weight gain in rats throughout the 10-week experimental period. The dramatic obesity which resulted from OVX occurs as a consequence of reduced energy expenditure and lipid metabolism in rats, and thus causes increased fatty deposits in the adipose tissue [28], [29]. But, the remarkable increase of body weight was partially suppressed after rats were exposed to PEMF. Therefore, these findings indicate that 10-week whole-body exposure to PEMF partially reversed OVX-induced weight gain via potentially preventing estrogen deficiency-induced decrease of energy expenditure in the adipose tissue.

Bone is composed of hierarchical bio-composite materials containing both inorganic compounds (hydroxyapatite) and organic compounds (collagen, proteoglycans and glycoproteins). The biomechanical strength of bone, as a critical indicator of bone fragility and fracture risk, is jointly determined by its inorganic compounds and organic compounds [30]. OVX is able to impair bone’s mechanical integrity and influence its capacity to resist fracture [31], [32]. In our present study, bones were mechanically tested under wet conditions, which resembled their physiological conditions and might help provide better prediction precision for the mechanical properties than the dry bone testing after dehydration [33]. Consistent with previous findings [31], [32], our results in the present study demonstrate that the characteristic parameters of biomechanical strength in the femur diaphysis, including extrinsic biomechanical properties (maximum load and energy absorption) and intrinsic mechanical properties (elastic modulus), were significantly reduced by OVX. Furthermore, in line with previous findings, our present data also reveal that OVX led to dramatic increase of the femoral cross-sectional moment of inertia [34], but did not exhibit significant impact on the femoral stiffness [35], [36]. However, the decrease of maximum load in the OVX rat femur was partially inhibited by PEMF, indicating increased ultimate strength of bone to resist fractures. Moreover, 10-week PEMF stimulation significantly reversed OVX-induced deterioration of the elastic modulus of bone, which reveals potential significantly improved biomechanical properties of the cortical bone.

Sharing similarities with postmenopausal women, OVX rats experience significant bone loss due to estrogen deficiency, which mainly results from trabecular bone loss [37]. Therefore, the trabecular bone microarchitecture is considered to be a good predictor of OVX-induced bone loss and bone quality deterioration [38]. As noted previously in the MicroCT analysis, normal trabecular bone microarchitecture dramatically deteriorates after OVX [39], [40]. In accordance with these findings, our results also demonstrate notable trabecular bone deterioration, evidenced by decreased BMD, Tb.N, Tb.Th and BV/TV, and increased Tb.Sp. Moreover, OVX increased the SMI of the trabecular bone, demonstrating a potentially much less plate-like structure [41], which is consistent with deteriorated bone strength induced by OVX. Further observations demonstrate that PEMF partially reversed OVX-induced decrease of trabecular bone mass, deterioration of trabecular bone microstructure and increase of rod-like structure. Together, our findings of MicroCT analysis indicate that OVX-induced trabecular bone deteriorative process is able to be partially reversed by 10-week whole-body PEMF exposure.

Canonical Wnt signaling is the key regulator of bone mass, bone modeling and remodeling, and bone homeostasis [21], [42]. Extracellular Wnt proteins can bind to the Frizzled and LRP5/6 co-receptors on the cell membrane, and thus lead to the stabilization of β-catenin in the cytoplasm and promote more Wnt-targeted gene transcription in the cell nucleus. Activation of canonical Wnt signaling can promote osteoblastogenesis and osteobalst/osteocyte activity, as well as inhibit osteoclastic bone resorption indirectly. Strong evidence for the essential role of canonical Wnt signaling is derived from the osteoporosis phenotype displayed in Wnt, LRP5 or β-catenin gene knockout mice [43], [44], [45]. In line with previous findings [46], the overall mRNA expressions of Wnt, LRP5 and β-catenin within the tibiae were elevated in OVX rats in the present study, revealing high bone metabolism activities under OVX conditions. The major gene expressions in the canonical Wnt signaling pathway, including Wnt, LRP5 and β-catenin, were all significantly increased after 10-week PEMF treatment, demonstrating the potential activation of Wnt/LRP5/β-catenin signaling pathway by PEMF exposure. Coupled with the results that PEMF stimulation significantly improved bone mass and mechanical strength in OVX rats, our findings indicate that Wnt/LRP5/β-catenin signaling has been implicated in regulating PEMF-induced attenuation of bone loss in OVX rats.

RANKL-RANK signaling has been recognized as an essential pathway in the regulation of bone remodeling via modulating osteoclast development and activation [22], [25]. RANKL, as the cytokine mainly secreted by osteoblasts and osteocytes in bone, can specifically bind with RANK on the cell membrane of osteoclasts to activate a range of downstream intracellular signaling pathway and hence promote osteoclastogenesis. Studies have shown that mice with deficiency in either RANKL or RANK displayed severe osteopetrosis and profound defect in bone resorption and remodeling [24], [47]. Therefore, RANKL-RANK signaling that is specific to osteoclasts has been widely acknowledged as a potentially promising new treatment target for osteoporosis in clinics [22]. In the present study, our findings demonstrate that the overall gene expressions of either RANK or RANKL in the skeleton of OVX rats were not obviously changed after 10-week PEMF exposure, indicating that PEMF did not exert its regulatory effects on RANKL-RANK signaling to modulate bone remodeling. Therefore, our present findings reveal that the RANKL-RANK signaling pathway might not be the principle mechanism by which PEMF improved bone microstructure and mechanical strength in OVX rats.

In conclusion, the present study clearly demonstrates that PEMF stimulation is able to partially prevent estrogen deficiency-induced bone loss in OVX rats, evidenced by the results of biomechanical testing and MicroCT analysis. The RT-PCR results further demonstrate that PEMF exposure can increase the gene expressions in Wnt/LRP5/β-catenin signaling, but not in RANKL-RANK signaling. Our findings highlight that PEMF modulated bone microarchitecture and strength via potential Wnt/LRP5/β-catenin signaling. Understanding the *in vivo* anti-osteoporotic mechanism of PEMF may be helpful for the efficient and scientific use of PEMF stimulation on osteopenia/osteoporosis in clinics.
